# Beyond the EPR: Complementary roles of the hospital-wide electronic health record and clinical departmental systems

**DOI:** 10.1186/1472-6947-9-29

**Published:** 2009-06-12

**Authors:** Eivind Vedvik, Aksel H Tjora, Arild Faxvaag

**Affiliations:** 1 Norwegian Research Centre for Electronic Patient Records, Faculty of medicine Norwegian University of Science and Technology, Medical-technical research centre, N-7489 Trondheim, Norway; 2 Department of Sociology and Political science, Norwegian University of Science and Technology, Trondheim, Norway

## Abstract

**Background:**

Many hospital departments have implemented small clinical departmental systems (CDSs) to collect and use patient data for documentation as well as for other department-specific purposes. As hospitals are implementing institution-wide electronic patient records (EPRs), the EPR is thought to be integrated with, and gradually substitute the smaller systems. Many EPR systems however fail to support important clinical workflows. Also, successful integration of systems has proven hard to achieve. As a result, CDSs are still in widespread use. This study was conducted to see which tasks are supported by CDSs and to compare this to the support offered by the EPR.

**Methods:**

Semi-structured interviews with users of 16 clinicians using 15 different clinical departmental systems (CDS) at a Medium-sized University hospital in Norway. Inductive analysis of transcriptions from the audio taped interviews.

**Results:**

The roles of CDSs were complementary to those of the hospital-wide EPR system. The use of structured patient data was a characteristic feature. This facilitated quality development and supervision, tasks that were poorly supported by the EPR system. The structuring of the data also improved filtering of information to better support clinical decision-making. Because of the high value of the structured patient data, the users put much effort in maintaining their integrity and representativeness. Employees from the departments were also engaged in the funding, development, implementation and maintenance of the systems.

**Conclusion:**

Clinical departmental systems are vital to the activities of a clinical hospital department. The development, implementation and clinical use of such systems can be seen as bottom-up, user-driven innovations.

## Background

Clinical departmental systems (CDSs) are small hospital information systems that are tailored to the information needs of one or a few clinical departments [1]. Despite many attempts to create hospital information system architectures that allows for the integration of such systems into larger hospital information systems [2-5], there is no standardized, simple approach to successful integration [6-8]. As of today, many clinical departmental systems therefore remain to be integrated. In 2004/2005, we surveyed clinical departments of a university hospital for the presence of clinical departmental systems and found 60 different systems that not were integrated with the hospital-wide electronic patient record (EPR) system [9]. 19 of these had been introduced after the implementation of the hospital-wide electronic patient record in 1999/2000. It is widely acknowledged that there is a huge demand for EPR systems that are better at supporting clinical workflow [10]. Assuming that CDSs were better at supporting important workflows at clinical departments, the purpose of this study was to further explore why clinician's demand for clinical departmental systems still existed 5 years after the implementation of an EPR system. Which particular CDS-functions do the users perceive as valuable, and how do the CDSs compare to the hospital-wide EPR system?

## Methods

### Selection of case hospital and information systems

St.Olavs Hospital is a medium sized (964 beds) general university hospital located in Trondheim, Norway. It serves as a community hospital for a local population of 280.000 and as a specialized hospital for all inhabitants of Central Norway (630.000). At the time of the investigation, the hospital had 17 departments. The core patient information systems in the hospital include a patient-administrative system (PAS), an image archiving and -communication system (PACS), a laboratory information system (LIS) and an electronic health record (EPR) system (DOCULIVE). The case was selected on basis on convenience, being close to the research centre (NSEP) to which the authors are affiliated.

In an earlier study of CDSs in the same hospital [9], 60 different CDSs were selected for a quantitative analysis of system use and functionality. During this study, the first author (Vedvik) observed that a subgroup of the systems provided crucial support for specific tasks, compared to the "average" CDS. On basis of these characteristics, 15 such systems were selected for a qualitative follow-up study reported here. Data about the systems are presented in table 1.

**Table 1:** Clinical Departmental systems

#	System	Vendor/Developer	Principal function	Department used
2	Stomy- follow-up	Former employee at the department together with Hospital IT-department	Follow-up of patients with ileostomy or colostomy	Dept. of Surgery
4	Datamed Føde ([Birth])	Former employee at the department together with local IT-company that later was acquired by a larger company	Management of birth data	Dept of Obstetrics
6	OpPlan	Employee at Dept of orthopaedics/Hospital IT-department	Operation room management	Operation room
8	Cytodose	Clinsoft AS http://www.clinsoft.no	CPOE system for prescribing drugs to cancer patients	Dept of Oncology
10	Shire	Asker Data AS http://www.askerdata.no	System for follow-up of patients with genetic diseases	Dept. of Medical genetics
12	Sympathy	TietoEnator Corporation http://www.tieto.com	Laboratory system	Dept of Pathology
15–18	N.O.A.H., Hast dB, Audera, EZ-screen.	Hast dB: Employees at the department in collaboration with hospital IT-department The others: Vendor of Technical equipment in use at the departiment	Management of patients with ear diseases and hearing aids.	Dept of Ear, Nose and Throat
22	Slagbasen ([StrokeBase])	Hospital IT-department	Management of patients with stroke	Stroke care unit
36	Hjerteinfarktregisteret ([Myocardial infarction registry])	Hospital IT-department	Registry for securing quality of care given to patients with acute myocardial infarction	Dept. of Cardiology
39	Anesthesia	Former employee at the anesthesia department	Quality control of anesthesia procedures	Dept. of Anesthesia
42	Gastro journal	Employees at the department in collaboration with Local IT-company	Documentation of endoscopic procedures	Dept. of Gastroenterology
43	Cardio	Employee at the department (first versjon), later versions developed by Hospital IT-department	Documentation of cardiologic ultrasound procedures	Dept. of Cardiology
47	HjerteRytme ([Hearth Rhythm])	Vendor of Technical equipment in use at the departiment	Documentation of the use of pacemakers and defibrillators	Dept. of Cardiology
48	SfB ([System for Infertility])	Small local IT-company	Management of infertility and assisted reproduction	Dept of Gynaecology
57	Visupac	Vendor of Technical equipment in use at the departiment	Record system for	Dept of Ophthalmology

### Data by semi-structured interviews

This study is based on data generated by using semi-structured interviews [11]. in which one experienced user per system was expected to elaborate on experiences related to qualities and aspects with the system. For one of the 15 systems, both a clinician and a clerk were interviewed, due to the differentiated tasks supported by this particular system. Hence, a total of 16 informants (one clerk, six physicians, nine nurses) were interviewed in the study. We do not, in this article, aim to generate general knowledge about all kinds of CDSs, but rather to explore how clinicians have experienced using the 15 systems selected, and thereby develop knowledge of the longevity of application of some systems. Our aim in this part of the project has been to identify common characteristics with these selected systems, rather than variations between users' experiences with each system. Therefore, only one informant has been interviewed for each system (and two for one of the systems). The selection of research subjects has been strategic on two levels, selecting systems that are of major clinical importance, and selecting one informant for each system, who is an experienced user who have been involved for a long time, often with systems development and adjustments, as well as use.

The following topics were included in the interview guide: (a) background for the initiative to develop and/or purchase a CDS, (b) history of development and implementation of a first version, (c) the role(s) and specific function(s) of the CDS in the department, (d) particularly important features of CDSs that might explain their longevity in the department, (e) plans, wishes or ideas for further development of the CDS, and (f) challenges with regard to integration with the hospital's EPR system and other systems. Both the first author and sociologist research assistant participated in the interview sessions, which were all audio-recorded. The interview subject was free to choose the course in each topic. Each interview lasted approximately one hour. All interviews were fully transcribed ad verbatim and imported into HyperRESEARCH 2.5 software for qualitative analysis.

### Inductive analysis of interview data

All interviews were analysed by inductively identifying text segments (sentences or paragraphs) and marking those with thematic 'codes' that highlight certain aspects of the empirical material, inspired by a grounded theory approach [12]. During this first analysis, the meanings and definitions of each code were continuously updated as new aspects came up in the material. Also, new codes were introduced as new topics were being discussed. Finally, this process resulted in 82 codes with distinctive meanings. In sum, the codes represent labels for the different statements throughout the interview material. Using the HyperRESEARCH software for qualitative data analysis, we generated reports sorted by code and linking interview extracts from the interviews. On basis of these reports we sorted out five main themes, to answer the research questions in this article.

## Results

Five main themes sum up the relevant issues as reported during the interviews:

a) Using CDSs to aid decision-making, information overview, and documentation

b) Using CDSs for quality control, learning, and research purposes

c) The life-cycle of CDSs

d) Data security and confidentiality of CDSs

e) CDSs for coordination of clinical tasks

The details of the results will be structured on basis of these themes.

### The CDS for decision-making, information overview, and documentation

Information systems may assist in decision-making by providing clever overviews of patients or by offering regular rule-based decision-support tools. Many respondents emphasized the benefits of having instantaneous access to patient data electronically, instead of having to look up in paper records:

"When the patients are on the phone we just start the program, type our initials and the date and [look up] who saw the patient at the last encounter six months ago etc, then we are updated at once on the issue and what has happened" (CDS-2).

Others emphasized the importance of having a system that not overwhelmed the user with patient data (which seems to be a problems with the EPR for some users) but instead only presented the data of relevance to the current problem. A CDS might for example generate a summary specifically designed for one disease or diagnosis.

"I think that the summary that lies in [the system] on patients with gastrointestinal disease, I regard as very, very useful. " (CDS-42).

Other clinicians argued that the system presented a better overview of the patient and consequently supported better decisions:

"Since you can compare results over time and follow the development [of the disease], the issue is not only whether or not the disease has progressed [to the worse] but also how fast the disease has progressed, and [getting this overview] is clearly more easy when you have these data in an database. So it obviously improves the quality of the care." (CDS-43).

When it came to making a report of what had happened with the patient during an encounter, some preferred to use a CDS rather than the EPR:

" [In the CDS] all is written in one page, the important parts of the history, the examination and the conclusion, so we have one page that gives us the essence. And this is a very handy document, you know."(CDS-42)

"We document nothing except what we write in the [CDS]. The report is then printed and archived in the paper based medical record. We almost never use the [EPR]. Instead we attach the report from the [CDS]." (CDS-42).

Others took the job of duplicating data recorded in the CDS:

"When we have made the report in the [clinical departmental system] we highlight it, and make a copy and then inserts it into the [hospital-wide electronic] medical record. " (CDS-2).

Many saw the CDS as a relevant expansion of the general medical record:

" [The CDS] may be classified as a record system, but the level of detail is beyond that of what usually gets documented [in the general medical record]. ..but the core information [of the CDS] corresponds to what belong in the general medical record." (CDS-22).

It is a general impression that CDSs are more thoroughly developed as integrated in the clinicians' practice, not at least with a more immediate interface accessing those elements of data that are relevant for department-specific clinical work.

### The CDS for quality control, learning and research

The informants reported that they conducted a plethora of 'quality control acts' with use of the system. For some, the quality of the data in the system was an important issue. This had led to routines ensuring the correctness of the recorded data and giving feedback to the registrants when erroneous recordings were discovered.

"..and what I have done is to check the reports for errors and give feedback [to the users] that this [registration detail] most probably is an error" (CDS-36).

Others had conducted formal evaluations of the quality of the data in the system and found that it was good enough:

"We have done some validations on [the system] and compared [the data in the system] with all other information sources to check if the data [in the system] are correct, and on an overall basis, the [system] is more reliable than the medical records.." (CDS-22).

Other informants (CDS15-18) also highlighted how the differences in data quality when comparing the CDS with the general EPR, to point out that if the data were different in these systems, they would trust the data in the CDS rather than those in the paper medical record.

Data that are archived in clinical departmental systems originate from clinical work. Not surprisingly, many informants reported on using the data to control and improve the overall quality of the work. For some informants, the use CDSs to ensure [the quality of] the treatment of the patients was an imperative. The perspective was not only on the treatment of the patients as individuals, but also on the treatment of the patients as a group, the entire "patient population" (CDS-36).

".. for instance in [Town] the nurses have collected data by use of the system, and then they became aware that the time that elapses from the patient enters the hospital until they are provided the therapy.. thrombolysis for instance, which must be administered pretty early in the course of the disease, the time that actually elapsed [before the patient was given therapy] was way too long, and then they have met with the staff at the hospital emergency department and asked them why, what happened [with the patient while he was] there and why it took so long time. So [the staff at the hospital] woke up then." (CDS-36)

Many informants reported to query the system for statistical reports that subsequently would be used within the department or in discussions with other departments.

"...we can say that we have given so many thousand [patients] anesthesia and spent so many hours on each department, of which 60% has been at nighttime ... To have such data is rather useful when we have resource discussions with other departments ... if they feel they always get too little [support from the anesthesia department] and we can show that they got more [this year] than last year." (CDS-39).

Others reported to use the CDS to generate reports for the hospital administration or the health authorities:

"Frequently, we are asked to generate some kind of report. The request may come from the hospital administration, the national medical association or a national project." (CDS-42)

One system was used to inform colleagues at another hospital by sharing information on best practice:

"It has happened several times that people from the national hospital have contacted us saying that they planned to give a cure that they haven't used before and then asked us of we could send [information from CDS] electronically" (CDS-8).

Reports from the systems were also utilized for supervision of junior colleagues and in the education of residents (CDS-47).

To summarize, data from a CDS were both used as feedback to those clinicians who reported the data, and to the department as a whole, as a basis for learning and quality improvement. Our study clearly shows that the various departmental systems are tightly integrated with quality control, learning and research workflows at the departments, and make a difference for the departments as a whole, for clinical practice and for the particular methods, diseases and patients that is provided care at the department.

### The life cycle of CDSs

Tailoring a system to the needs of the users demands an interaction between the users and the information system experts and/or developers. Some CDSs were commissioned from an IT vendor that developed the system in close collaboration with the department. Others were developed by the hospital-internal IT department. Having good access to the systems developers was considered beneficial, for instance through "a hotline to the IT department" (CDS-36). Computer-savvy employees at the ward had developed some of the systems. In one case, the user-developer constructed the system in the anticipation that the system would become a product and give reimbursement from sales to other hospitals:

"Those who made it was not IT-people; the initiative came from an otorhinolaryngologist that was interested in IT, I guess he saw an opportunity to make money " (CDS-4).

Having the developer within the department might ensure a tight interaction between the developer and the user. However, having the system developed by an employee at the department also had its downside, for instance losing support for the system when the developer/clinician no longer worked at the department. Some systems were no longer maintained by an IT developer, a company or the IT-department, and thus had become "antique" (CDS-4).

This could be due to lack of funding for an upgrade. A system would be developed as an "interim solution that have become permanent, because of lack of funding for a new upgrade" (CDS-43). In other cases, the department might "have jumped off the *upgrade spiral *and kept the old solution" (CDS-39). Other systems ended up in a stagnant position because people or companies had disappeared, for instance being "bought by another company" (CDS-4), thus losing a committing relation between clinic and developer. Such situations would have negative impacts on the value and perceived usefulness of the system. One informant reported that some parts of the system had ceased to function. One of the orphaned systems were in a particularly marginal situation:

"The others who knew about the program that we have used no longer work here, so now only I am left" (CDS-39).

Some informants reported of a different (or changed) attitude among the employees towards a CDS and that this had implications for interaction between the users and the system. One informant reported having problems getting the doctors to learn how to use the system. The doctors did not show up when a training course was arranged. Others reported of slow adaptation by the users and that this had a negative impact of their utility of the functions that the system offered:

" [It took a] long time before the users knew how to use the system. [..] After 4 years we have discovered some new buttons" (CDS-10).

A major problem seems to be the very fragile foundation of some CDSs in departments. Lack of continued commitment from clinicians and/or developers is a serious threat to the prolonged value of a CDS.

### Data security and confidentiality

One CDS in the study was developed for archiving highly confidential patient data, which was not allowed to be stored in the general hospital-wide patient record system. Another system was implemented with a feature that made it possible not to disclose the identity of the patient when this was not necessary:

"Earlier, you know, when the operating theatre schedule was a paper document, between 70 and 80 paper copies were distributed and put in the pockets [of surgeons' coats] everywhere. Now, we can print schedules that do not have the names of the patients" (CDS-6).

But the respondents also mentioned information security breaches, for example not being able to find patients that should have been in the systems. Downtime was one possible explanation for this problem. Not having access to the CDS when seeing patients at other departments was also mentioned as a problem.

### Coordination of clinical tasks

Some systems were used for planning and coordination of work in the ward. One system was used to identify bottlenecks in the production line (CDS-12) and another was designed simply to plan the use of a particularly scarce or expensive resource at the department (CDS-6, CDS-47). Interestingly enough, for a CDS, these systems would also be used to coordinate care across hospital boundaries (CDS-8).

## Discussion

In this article we have applied semi-structured interviews of health personnel to identify experiences with the applications of 15 different CDSs at a University Hospital in Norway. By analysing interview data inductively, we are able to understand the many roles of these systems at the clinical wards and why some CDSs still prevail, more than five years after an EPR system was implemented throughout the hospital. The fifteen CDSs selected for this study provided functionality that made them essential for the daily clinical work, for quality improvement, for supervision, or for clinical research. According to the clinicians, the hospital-wide EPR system lacked many of these features. In situations where the respondents could choose between a CDS and the EPR, they highlighted the domain-specific features of the CDS (e.g. problem-specific recording and presentation of patient data) as reasons for preferring the CDS. In general, we conclude that the CDSs both substitutes and complement the EPR system in assisting clinical work.

Our discussion here is structured around the issues of, first, how CDSs and the EPR systems are perceived and used differently, and second, how we perceive CDSs as related to user-driven innovation.

### Preferring the CDS to the EPR

Compared to the hospital-wide EPR system, the CDSs were able to support a wider spectrum of clinical and local administrative tasks. CDSs were preferred to the EPR system because they aimed at processing, storing and presenting only relevant patient data and thereby giving better overview over the situation of the patient. Some CDSs seem to possess such functions whereas the EPR-system does not.

#### Functionality

The lack of such functionality in EPR systems has partly to do with EPR systems' application of free text presentation [13]. In such systems, patient data are archived by the EPR system but there is no integrating structure to enable EPR data to be integrated with the CDS. Instead, they are, as free text, optimized for being read and comprehended by "human information processors". They are also optimized for the preparation of paper copies of the text and for documenting in the legacy paper-based medical record. However, as hospitals increasingly take the steps to completely replacing the paper-based medical record with an EPR, the requirement to replicate the EPR on paper is about to become obsolete. In this perspective, the results from this investigation may provide clues to which functionality must be implemented in an EPR system to provide clinicians with a better alternative than their CDSs. We consider some CDSs to illustrate of such functionality that should inspire requirement engineers and EPR system developers.

#### CDS as quality register

Another core function reported for many CDSs was as repositories of patient data that can be analyzed to determine the outcome of comparable health care acts or the patients as a group. A quality register may be defined as a registry that contains comparable patient data that stem from encounters between patients and health care providers and that is archived so that the meaning of the data is preserved and the data can be analyzed by a computer program. Quality registers may take the shape of disease registries or registries for patients exposed to a particular healthcare intervention technique. Quality registries may have the aim of being population based, i.e. to contain data from all relevant patients in a given geographic area or be restricted to data from patients in contact with group of healthcare providers. CDSs were used to analyze and subsequently improve the outcome of healthcare acts conducted at the department, to generate activity reports demanded by the hospital administration and to evaluate the quality of the work of trainees for use when supervising them. Taken together, our data clearly illustrate that CDSs acts as institution-bound quality registries that satisfies clinicians desire for "building measurement and data collection into medical practice"[14].

#### Better CDS data quality

Many informants expressed a sense of ownership to the data contained in the CDS. As such they also felt responsible for the quality and integrity of the data. We believe this attitude has to do with the relatively broader set of healthcare functions that can be enacted with use of the data in a CDS. Since more functions can be performed with use of the data, increased efforts were taken to keep the data reliable. The departments also deposited patient data in the hospital-wide EPR system. The informants however revealed a slightly different attitude towards these data and also reported to care less for their integrity. Accordingly, the informants regarded patient data archived in the hospital-wide EPR system as less polite compared with those in the CDS.

### CDSs as the result of user-driven innovation

The informants reported to be engaged in roles beyond that of a regular user of the information systems. Some CDSs were the result of initiatives from healthcare personnel at the ward. Typically, personnel had engaged with a local company or an independent developer and tailor-buildt the system to the needs of the department. The informants also were concerned with data quality, funding and maintenance issues. The emergence and implementation of CDSs might be characterized as instances of user-driven innovation [15]. User-driven innovation are the situations where the users of a service or product develop ideas as to how the product could be improved and thereafter contribute to design or manufacturing of an improved version of the product. User-driven innovation has been described in scientific communities [16], software development [17], drug therapy [18] and many other domains [19]. According to Von Hippel [19], users regularly find mismatches between features of existing products and their particular needs. Because some users have deeper insight in the need that the product is supposed to satisfy and the products' context of use, users tend to engage in modifying and thereby improving the products. Von Hippel characterizes users that engage in product development or product modification processes as *lead users *because they are at the forefront of a market trend [19]. As the quality and availability of software and hardware tools for innovation has improved and the Internet has brought user innovators together, user-driven innovation is being *democratized *[19]. Increasingly, manufacturing companies must re-organize their own innovation processes to incorporate and further improve on innovations created by lead users.

Our case illustrates that user-driven innovation also occurs in the domain of clinical information systems and that this has contributed to systems with highly valuable features. Some CDSs were the realization of the clinicians' ideas on how to collect and visualize patient data, and how to re-use the data in processes not directly related to the care of the individual patient. While user-driven innovation of clinical information systems may result in systems that get to occupy important roles at the clinical departments, we also uncovered a number of problems with this approach. In our case, the manufacturing companies were small and tended to disappear or being acquired by other, larger companies. We found that some CDSs no longer were maintained, either due to lack of funding or because the staff who once engaged in constructing and implementing the system no longer were employed at the ward. Some of the CDSs were probably not constructed with the needs for integration and upgrading in mind. CDSs on technologically outdated platforms were still preserved because of the unique value of the data contained in them. In other domains, it has been reported that manufacturers improve on their products by incorporating features first invented by lead users [19]. Since interviewing representatives from manufacturing companies not was part of our study, we cannot decide on whether manufacturers of CDSs organize their product development processes to benefit from user innovations. Neither of our user informants however reported of such events.

Our study was conducted at a time when an integrated, hospital-wide EPR system was meant to incorporate most of the patient-related data at the hospital and gradually eliminate the need for specialized CDSs. What is of relevance to user-driven innovation and product improvement is that our informants complained of not having any contact with the manufacturers of this new system and therefore not being able to influence on its design and features.

In the analysis of our data, we must carefully take into consideration that our informants were recruited as enthusiasts (at various levels) towards the different CDSs. This limits the generalisability of our study, not at least when it comes to comparing the EPR system and the CDSs. The deep engagement of some informants in the development and maintenance of the CDSs makes them, as users, developers, or both, generally sympathetic to "their" systems. These clinicians' subjective experienced-based comparison between a CDS and the hospital's EPR system must necessarily result in favour for the CDS, both because clinicians may not apply all functionalities of the EPR [20] and because of a *not-invented-here syndrome*. Having said this, our informants have been very detailed on the assessment of CDS and EPR system functionalities. Such detailed descriptions have reduced the methodological challenge of potentially strategically motivated unbalances, and strengthened reliability, despite the necessary consideration that must be taken towards enthusiast loyalty.

### Implications for EPR system requirements

Users of some CDSs believed that their CDS could be integrated in the hospital-wide EPR system in the future. The main concern among most users, however, was the fear of loss of valuable functionality if their CDS was to be phased out and the data integrated into the EPR. The users also feared of losing influence and control over the development. Other CDSs, however, are of such nature that neither their users nor hospital administration today see any reason why these systems should become an integral part of the EPR system.

We have not, as part of our project, studied in technical matters how the CDSs and the EPR system could or should be integrated. What we find is that CDS users are concerned about the maintenance (or conservation) of the CDS as the EPR system is implemented across the hospital. While the EPR system seems to be introduced on basis of an administrative rationality (top-down), the CDSs represent a technologically mediated departmental clinical rationality. Hence, the CDSs have internalised a professional awareness [21] that the clinicians are not able to identify as part of the hospital-wide EPR system. Even though a sound development of hospital information systems would include functionality from the CDSs being integrated in the EPR system, such integration represents less departmental control over clinically relevant information. Studies within CSCW (computer-supported cooperative work) [7,8,22,23] have emphasised that technologies are often integrated in the social domain rather than technically, and that redundancy is not only a problem, but also a source of security. In this view, a number of CDSs may live well together with the EPR. However, within such a strategy, increasing clinically essential functionality of the EPR will suffer.

## Conclusion

We have depicted the many roles of clinical departmental information systems in a Norwegian university hospital and argued that such systems are the results of user-driven innovation. In a hospital management era characterized by a belief in the value of large, centralized, and interdepartmentally integrated EPR systems, vendors of such EPR systems should re-organize their product development processes to incorporate features and ideas developed by innovative lead users.

## Competing interests

The authors declare that they have no competing interests.

## Authors' contributions

AF, EV and AHT designed the study. EV conducted the interviews and transcribed the data. AF, EV and AHT analyzed the data and wrote the paper. All authors read and approved the final manuscript.

## Pre-publication history

The pre-publication history for this paper can be accessed here:

http://www.biomedcentral.com/1472-6947/9/29/prepub

## References

[B1] Van Bemmel J Musen MA Handbook of Medical Informatics 1997 New York: Springer Verlag

[B2] Xu Y Sauquet D Zapletal E Lemaitre D Degoulet P Integration of medical applications: the 'mediator service' of the SynEx platform Int J Med Inform 2000 58–59 157 166 10.1016/s1386-5056(00)00084-8 10978918

[B3] Altmann U Tafazzoli AG Katz FR Dudeck J XML-based application interface services – a method to enhance integrability of disease specific systems Int J Med Inform 2002 68 27 37 12467788 10.1016/s1386-5056(02)00061-8

[B4] Grimson W Berry D Grimson J Federated healthcare record server – the Synapses paradigm Int J Med Inform 1998 52 3 27 9848399 10.1016/s1386-5056(98)00121-x

[B5] Lenz R Blaser R Kuhn KA Hospital information systems: chances and obstacles on the way to integration Stud Health Technol Inform 1999 68 25 30 10724881

[B6] Cruz-Correia RJ Vieira-Marques PM Ferreira AM Almeida FC Wyatt JC Costa-Pereira AM Reviewing the integration of patient data: how systems are evolving in practice to meet patient needs BMC Med Inform Decis Mak 2007 7 14 17565667 10.1186/1472-6947-7-14 PMC1919361

[B7] Ellingsen G Monteiro E A Patchwork Planet Integration and Cooperation in Hospitals Computer supported Cooperative Work 2003 12 71 95

[B8] Monteiro E Integrating health information systems: a critical appraisal Methods Inf Med 2003 42 428 432 14534645

[B9] Vedvik E Faxvaag A The fate of clinical department systems at the dawn of hospital-wide electronic health records in a Norwegian university hospital Stud Health Technol Inform 2006 124 298 303 17108540

[B10] Committee on Data Standards for Patient Safety Key Capabilities of an Electronic Health Record System 2003 Washington DC: The National Academies Press 25057672

[B11] Kvale S Brinkmann S InterViews: Learning the Craft of Qualitative Research Interviewing 2008 London: SAGE

[B12] BG G AL S The discovery of grounded theory 1967 New York: Aldine de Gruyter

[B13] Lium J Faxvaag A Tjora A No paper, but the same routines: a qualitative exploration of experiences in two Norwegian hospitals deprived of the paper based medical record BMC Med Inform Decis Mak 2008 8 2 18186935 10.1186/1472-6947-8-2 PMC2245928

[B14] Nelson EC Splaine ME Batalden PB Plume SK Building measurement and data collection into medical practice Ann Intern Med 1998 128 460 466 9499330 10.7326/0003-4819-128-6-199803150-00007

[B15] von Hippel E The sources of innovation 1988 New York: Oxford University Press

[B16] Riggs W von Hippel E Incentives to innovate and the sources of innovation: the case of scientific instruments Research Policy 1994 23 459 469

[B17] Lakhani KR von Hippel E How open source software works: "free" user-to-user assistance Research Policy 2003 32 923 943

[B18] Demonaco H Ali A von H Eric The major role of clinicians in the discovery of off-label drug therapies Pharmacotherapy 2006 26 323 332 16503712 10.1592/phco.26.3.323

[B19] von Hippel E Democratizing Innovation 2005 Boston: MIT Press

[B20] Laerum H Ellingsen G Faxvaag A Doctors' use of electronic medical records systems in hospitals: cross sectional survey BMJ 2001 323 1344 1348 11739222 10.1136/bmj.323.7325.1344 PMC60674

[B21] Tjora AH Scambler G Square pegs in round holes: information systems, hospitals and the significance of contextual awareness Soc Sci Med 2009 68 3 519 525 19054601 10.1016/j.socscimed.2008.11.005

[B22] Cabitza F When once is not enough: the role of redundancy in a hospital ward setting GROUP '05: Proceedings of the 2005 international ACM SIGGROUP conference on Supporting group work; 2005 2005 Sanibel Island, Florida USA. ACM 158 167

[B23] Tjora AH Maintaining Redundancy on the Coordination of Medical Emergencies 2004 Chicago: Sheridan printing Company

